# Measuring fear: Association among different measures of fear learning.

**DOI:** 10.1016/j.jbtep.2020.101618

**Published:** 2021-03

**Authors:** Elena Constantinou, Kirstin L. Purves, Thomas McGregor, Kathryn J. Lester, Tom J. Barry, Michael Treanor, Michelle G. Craske, Thalia C. Eley

**Affiliations:** a Social, Genetic and Developmental Psychiatry Centre, Institute of Psychiatry, Psychology & Neuroscience, King's College London, London, UK; b University of Sussex, School of Psychology, Brighton, UK; c Experimental Psychopathology Lab, Department of Psychology, The University of Hong Kong, Hong Kong, China; d Department of Psychology, Institute of Psychiatry, Psychology & Neuroscience, King's College London, London, UK; e Department of Psychology, University of California, Los Angeles, CA, USA; f Department of Psychiatry and Biobehavioral Sciences, University of California, Los Angeles, CA, USA

**Keywords:** Differential fear conditioning, Skin conductance, US-expectancies, Affective ratings

## Abstract

**Background and objectives:**

Fear conditioning paradigms use various measures to assess learned fear, including autonomic arousal responses like skin conductance, and self-reports of both associative (US-expectancies) and evaluative (affective ratings) learning. The present study uses a dimensional approach to examine associations among fear indices directly.

**Methods:**

Seventy-three participants completed a differential fear conditioning experiment, during which a neutral stimulus (CS+) was paired with an electric shock (US), while another stimulus (CS-) was never paired with the shock (partially instructed fear acquisition). Ten minutes later, both stimuli were presented without any shocks (fear extinction). Skin conductance responses and US-expectancy ratings were recorded during each phase, while self-reported negative affect was assessed for each CS at the end of extinction.

**Results:**

Results showed a positive association among US-expectancy ratings and skin conductance responses during acquisition and early extinction. US-expectancy ratings during overall extinction were positively associated with post-extinction negative affect.

**Limitations:**

The lack of affective ratings post-acquisition may have obscured associations between associative and evaluative learning indices.

**Conclusions:**

Results provide evidence for the expected correspondence among different indices of associative fear learning. Findings emphasize the need for incorporating both associative and evaluative learning measures in fear conditioning paradigms.

## Highlights

• Fear conditioning studies use physiological and self-report measures to assess fear.
• We examined the association among these indices using a dimensional approach.
• Autonomic arousal was associated with US-expectancy ratings.
• US-expectancy ratings during extinction predicted post-extinction negative affect.
• Results support the need for both associative and evaluative indices of learned fear.

## 1 Introduction

Aversive classical conditioning offers an explanatory model for the development of anxiety disorders (Davey, 1992a; Mineka & Oehlberg, 2008; Watson & Rayner, 1920). According to this view, pathological anxiety can develop when an aversive event (e.g. motor vehicle collision) is associated with a previously innocuous stimulus (e.g. highway driving). Human fear conditioning paradigms investigate this associative process under controlled experimental conditions and have advanced our understanding of the aetiology (Bouton, Mineka, & Barlow, 2001; Mineka & Oehlberg, 2008) and treatment (Craske, Hermans, & Vansteenwegen, 2006; Craske, Treanor, Conway, Zbozinek, & Vervliet, 2014) of anxiety disorders. Such paradigms typically involve the pairing of a neutral stimulus with an innately aversive stimulus e.g. an electric shock or loud noise (unconditional stimulus, US), which automatically elicits a defence reflex (unconditional response; e.g. increased heart rate). After multiple pairings, the neutral stimulus becomes associated with the aversive stimulus (i.e. becomes a conditional stimulus, CS) and elicits a defence response in anticipation of the aversive stimulus (conditional response, CR).

Successful fear learning within these paradigms is indicated by the presence of various conditional responses to the CS. This study investigates the relationship among different outcome measures indicative of fear learning. Fear comprises multiple components including elevated physiological arousal, subjective experience of fear, and behavioural avoidance (Lang, 1968). Therefore, learned fear can be assessed using measures reflecting these different elements. Autonomic arousal indices are the most typical outcome measures assessed in human fear conditioning studies and absence of such responses is often considered to reflect non-learning (although various problems have been identified with this practice; Lonsdorf et al., 2019). Skin conductance response to the CS is the most commonly used autonomic signal, although others such as the fear-potentiated startle, heart rate or pupillary dilation are also used (see overview by Lonsdorf et al., 2017). Studies also typically assess the subjective experience of learned fear, with self-reports measuring either the fear/anxiety induced by a CS (Mertens et al., 2018) or two underlying processes involved in fear learning: a) evaluative learning, i.e. change in the perceived unpleasantness of the CS due to its pairing with the US (affective ratings; De Houwer, Thomas, & Baeyens, 2001) and b) associative learning, i.e. the learning of the contingency between US and CS (US-expectancy ratings; Davey, 1992b). Behavioural avoidance has been examined to a lesser degree (Blechert, Michael, Vriends, Margraf, & Wilhelm, 2007; Grillon, Baas, Cornwell, & Johnson, 2006; Pittig, Schulz, Craske, & Alpers, 2014).

The association among different outcome measures of fear learning has been a matter of theoretical debate as some theorists propose that all fear components stem from one common learning mechanism (single process models; Lipp & Purkis, 2005; Lovibond & Shanks, 2002), while others assume that associative and evaluative learning comprise two distinct levels of fear learning (dual-process models; Öhman & Mineka, 2001). A recent dual-process model posits that the subjective experience of fear emerges from a higher-order cognitive neural circuit and is separable from physiological and behavioural responses, which are controlled by a subcortical amygdala-centered network (LeDoux & Brown, 2017; LeDoux & Pine, 2016). This model has rejuvenated the discussion about what fear is and how its different components link to each other (Fanselow & Pennington, 2018).

These theoretical views make assumptions about the correspondence among outcome measures of fear learning with implications for our understanding of fear and its assessment. Most research findings indicate that conditional skin conductance responses to the CS emerge only when individuals report awareness of the CS-US contingency, providing support for a correspondence between autonomic arousal and self-reports of associative learning (e.g. Baer & Fuhrer, 1968; Biferno & Dawson, 1977; Dawson & Biferno, 1973; Dawson, Rissling, Schell, & Wilcox, 2007; Hamm & Vaitl, 1996; Lovibond, 1992; Lovibond, Davis, & O'Flaherty, 2000; Marinkovic, Schell, & Dawson, 1989; Purkis & Lipp, 2001). However, this correspondence is not observed with other physiological signals, like fear-potentiated startle (Hamm & Vaitl, 1996; Sevenster, Beckers, & Kindt, 2012, 2014; Soeter & Kindt, 2010, 2012; Weike, Schupp, & Hamm, 2007), suggesting that skin conductance specifically is a physiological index of associative learning. The relationship of skin conductance with evaluative learning is less clear as some studies indicate a correspondence of skin conductance with affective ratings (Dawson, Rissling, et al., 2007; Purkis & Lipp, 2001), but others failed to do so (Blechert, Michael, Williams, Purkis, & Wilhelm, 2008; Luck & Lipp, 2015).

As for the association between associative (CS-US contingency) and evaluative (affective change in CS) learning self-reports, research initially showed that the perceived unpleasantness of a CS is not associated with the learning of the CS-US contingency (De Houwer et al., 2001; Lovibond & Shanks, 2002), but opposite findings have also been reported (Hofmann, De Houwer, Perugini, Baeyens, & Crombez, 2010). One finding suggesting distinct evaluative and associative learning processes is that, unlike associative learning measures, evaluative learning self-reports (affective ratings) remain unaffected by extinction (Baeyens, Díaz, & Ruiz, 2005; Hermans, Crombez, Vansteenwegen, Baeyens, & Eelen, 2002; Luck & Lipp, 2015). However, some studies showed extinction effects for affective ratings when assessed trial-by-trial during extinction (online), rather than after extinction (Lipp, Oughton, & LeLievre, 2003). Blechert et al. (2008), though, failed to show extinction effects for online affective ratings. Thus, the extinction of affective ratings and its relation to the extinction of other measures remains unclear.

The inconsistencies in the literature and the ongoing theoretical debate indicate the need for further research to clarify the associations among outcome measures of fear learning. Research should incorporate associative and evaluative learning indices within the same paradigm, instead of comparing responses to quite different associative and evaluative conditioning procedures (Blechert et al., 2008). Furthermore, research has seldom looked at the direct correlations among measures. Previous studies investigated correspondence by examining whether the patterns of each individual response co-vary, or by using group mean analyses, e.g. comparing responses in contingency aware vs. unaware individuals (see Lovibond & Shanks, 2002 for the rationale). A dimensional/correlational approach could be more informative as it assesses the strength of associations among measures. Such empirical data can inform theoretical models of fear learning, but also the methodological design of fear conditioning studies (choice of outcome measures). Furthermore, assessing the strength of associations allows the investigation of possible mediators/moderators of these associations, e.g. anxiety-related traits (Beckers, Krypotos, Boddez, Effting, & Kindt, 2013). To examine individual differences in this context, a shift towards dimensional approaches is required (Lonsdorf & Merz, 2017).

The present study uses a correlational approach to examine the association between physiological (skin conductance responses; SCRs) and self-report (US-expectancy ratings) indices of associative learning during partially instructed fear acquisition and fear extinction. Additionally, it examines the association of these two measures with an evaluative learning index, namely post-extinction negative affect towards the CS. Participants completed a differential fear conditioning paradigm, during which a neutral image was paired with an aversive stimulus (CS+) and a second neutral image was not paired with an aversive stimulus (CS-; fear acquisition). After a 10min break, CS+ and CS- were presented again without any aversive stimulus (fear extinction). We recorded SCRs and online US-expectancy ratings throughout acquisition and extinction, and collected affective ratings for each CS at the end of extinction.

Based on previous findings, we expected a positive association between differential (CS+ minus CS-) SCRs and differential US-expectancy ratings (Dawson, Rissling, et al., 2007) during both acquisition and extinction. However, associations were expected mainly during those parts of the task where such conditional responses are typically observed, i.e. during acquisition and early extinction (in AB designs, Sjouwerman, Niehaus, & Lonsdorf, 2015). We also expected weak or no associations between differential SCRs during acquisition or extinction and differential post-extinction negative affect. This is based on previous findings (Blechert et al., 2008) suggesting SCRs and affective ratings show distinct response patterns during fear conditioning. However, US-expectancy ratings and affective ratings were expected to associate positively, since they are both self-report measures and prior research suggests they follow a similar pattern (Blechert et al., 2008; Lipp & Purkis, 2005; Purkis & Lipp, 2001). As for the strength of these associations, based on theoretical views suggesting that response systems are “loosely integrated” (Lang, 1971, p. 165), we expected moderate associations.

## 2 Methods

### 2.1 Participants

Young adults between 21 and 26 years old, with no history of seizures, neurological or cardiac conditions and no current anxiety medication use were recruited among university students and the local community. Eighty volunteers completed the experiment. The data from three participants were unusable and four participants were excluded as extreme outliers (see Supplemental Material for details). Thus, the final sample consisted of 73 participants (19 males, M_age_ = 23.22, SD_age_ = 1.32). All participants provided written informed consent and received a small monetary compensation for their time. The study was approved by the Research Ethics Subcommittee for Psychiatry, Nursing and Midwifery of King's College London (HR15/16–2349).

As the experiment is part of a larger project (see Purves et al., 2019), this sample size was chosen to have adequate power (80%) to detect a moderate correlation (0.30), which would indicate meaningful associations between individual differences in fear learning and anxiety. A post-hoc sensitivity analysis (using the G*Power software) indicated that with the current sample we have power >80% to detect medium-sized effects in the presented regression models (R^2^ = 0.16 for early/late analyses and 0.14 for overall means; Cohen, 1988).

### 2.2 Materials and apparatus

The fear conditioning task was administered using E-prime 2.0 (Psychology Software Tools, Pittsburgh, PA). Physiological data were collected using BIOPAC MP150 and Acqknowledge 4.4 data acquisition software (Biopac Systems Inc., Santa Barbara, CA).

The unconditional stimulus (US) was an electric shock (500 ms) produced by a constant voltage stimulator (STM200) coupled with a BIOPAC STM100c amplifier and delivered through two 11 mm Ag/AgCl electrodes placed on the outside of participants’ dominant wrist. The shock intensity was individually calibrated at the beginning of the session to be unpleasant, but not painful (see Supplemental for calibration description).

The conditional stimuli were two orange circles, one small (2 cm diameter) and one large (10 cm diameter), which served randomly as either CS+ or CS-. During each trial, one of the two circles was presented in the middle of the screen overlaid on a coloured image (960 × 720px) used to differentiate the context between fear acquisition (outdoors garden scene) and fear extinction (indoors living room scene). The context changed between acquisition and extinction since the two phases were part of an ABA renewal design (participants also underwent a fear renewal phase 24 h later not reported here).

### 2.3 Procedure

Upon arrival to the laboratory, participants were told that the experiment “involved looking at shapes on the screen and sometimes receiving an unpleasant but not painful electric shock” and that their task was “to learn when a shape will be followed by a shock”. After fitting the electrodes, the shock calibration procedure took place followed by the differential fear conditioning task.

#### 2.3.1 Baseline

Participants viewed the small and large circles and completed baseline affective ratings (see below). The two circles were presented in random order.

#### 2.3.2 Fear acquisition

**Fig. 1:**
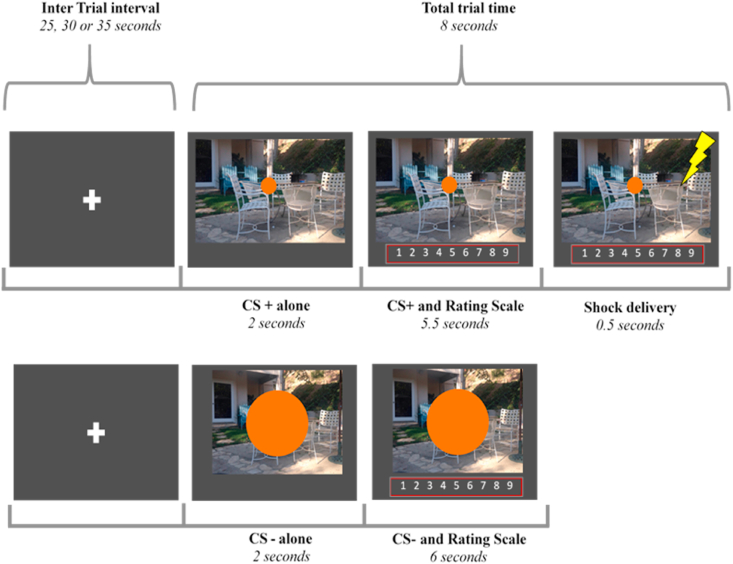
The structure of each fear conditioning trial (top row with a CS+, bottom row with a CS-). The unconditioned stimulus (electric shock) co-terminated with the CS+ on trials where it occurred. Note: The rating scale was presented along with anchors (1 = Certain No Shock, 5 = Uncertain, 9 = Certain Shock), which for practical reasons are not depicted here. Also, the rating scale was identical to the one used for baseline ratings, therefore participants had the chance to habituate to this stimulus prior to the first fear acquisition trial.

During fear acquisition, participants saw each CS 12 times (total 24 trials). Each trial lasted 8 s and was preceded by a variable duration inter-trial interval (ranging from 25 to 35 s; Fig. 1). One of the two CSs was paired with an electric shock at the chosen intensity for 9 out of 12 trials (75% reinforcement rate), thus becoming the CS+. The other circle was never paired with the shock (CS-). Trials were presented in a pseudo-randomized order (see Supplemental Material).

#### 2.3.3 Fear extinction

After a 10-min break, during which participants completed personality questionnaires not reported here, the fear extinction phase followed. During extinction, participants saw the CS+ and the CS- 24 times each (total 48 trials). Each trial had the same structure as in the fear acquisition phase, except that no electric shocks were administered.

#### 2.3.4 Post-extinction

After extinction, participants repeated the affective ratings for the two circles (in random order) and answered a contingency awareness question. Affective ratings were completed only after extinction and not beforehand, to avoid exposure to the CSs prior to the extinction phase and thus influencing participants’ responses during extinction (Lonsdorf et al., 2017).

### 2.4 Measures

#### 2.4.1 Physiological measures

Skin conductance was measured using two 11 mm Ag/AgCl electrodes placed on the palm of participants' non-dominant hand following standard procedures (Dawson, Schell, & Filion, 2007). The signal was recorded with a BIOPAC GSR100C transducer amplifier (gain = 20 μS/V, low-pass filter = 1 Hz, no high-pass filter) at 2000 Hz and smoothed over 1-s intervals offline. For each trial, we calculated the skin conductance response (SCR) by subtracting the average skin conductance level of the 2 s prior to each CS presentation from the peak during CS presentation (between 1 and 7 s of each 8 s trial; Pineles, Orr, & Orr, 2009; Zbozinek, Holmes, & Craske, 2015; Mertens et al., 2018). This method can detect differential conditioning effects (Pineles et al., 2009) and may be more appropriate than separating first and second interval responses in long acquisition phases, where response onset latency may shift over time (Jentsch, Wolf, & Merz, 2020). Each SCR was range-corrected using the individual's maximum skin conductance response to the electric shock (US; Lykken & Venables, 1971). After range-correction, negative SCRs were coded as zero and then each SCR was square root transformed to normalize the data. Finally, transformed SCRs were averaged for each CS and each phase of the experiment. Besides overall phase averages, we also calculated SCR averages for early and late acquisition and extinction to examine associations at specific parts of the task. Specifically, we divided each phase into thirds and used the first and last third as early and late parts respectively. For acquisition, the first four trials for each CS formed early acquisition and the last four late acquisition. For extinction, the first eight trials of each CS formed early extinction and the last eight late extinction. Physiological data pre-processing was done using Python and SCRs calculation using R (http://cran.r-project.org).

#### 2.4.2 Self-reports of associative learning

During each CS presentation, participants rated how much they expected to receive a shock (online US-expectancy ratings) using a 9-point Likert scale (1 = ‘Certain no shock’, 5 = ‘Uncertain’, 9 = ‘Certain shock’). Participants were instructed to respond by pressing the appropriate number on a keyboard placed underneath their dominant hand and keep their non-dominant hand stable (to avoid movement artifacts on the skin conductance measurement). After imputation of missing values,^1^ we calculated average US-expectancy ratings for CS+ and CS- for each phase overall, and for early/late parts of acquisition and extinction as detailed above. Participants also completed a contingency awareness question after extinction, i.e. if they noticed whether the shock was paired with a specific shape and which one. This indicated 66 contingency aware and 7 unaware individuals.

#### 2.4.3 Self-reports of evaluative learning

Participants provided affective ratings for each CS before acquisition (baseline) and at the end of extinction (post-extinction) using a 9-point Likert scale. They rated the extent to which each circle made them feel positive/negative affect (1 = Happy, pleased, satisfied, content, 9 = Unhappy, annoyed, despaired), fear (1 = Unafraid, safe, unconcerned, 9 = fearful, afraid) and anxious arousal (1 = Calm, sleepy, dull, unaroused, 9 = Anxious, aroused, excited, jittery). For all items, 5 was neutral and a higher rating indicated more negative affect. Missing data for these ratings were not imputed; rather participants were excluded from analyses if they missed more than one rating (1 participant missing all baseline ratings and 1 participant missing both baseline and post-extinction ratings were excluded). Exploratory factor analyses^2^ indicated that the three affective ratings loaded onto a single factor, therefore they were combined into a Negative Affect composite score (average of the three ratings) for each CS at baseline and at post-extinction.

### 2.5 Data analysis

For all measures, we calculated CS+/CS- differentials (a standard index for differential conditioning paradigms that taps into the specific effects of conditioning controlling for other non-associative processes like orienting, Lonsdorf et al., 2017), by subtracting the average SCR, US-expectancy ratings, and Negative Affect scores of the CS- from that calculated for the CS+. Differentials for overall acquisition and extinction and early/late parts of each phase were created. To confirm the expected fear conditioning effects on CS discrimination, two repeated measures ANOVAs with Phase (acquisition/extinction) and Time (early/late) as within-subject factors were performed for differential SCRs and US-expectancy ratings. For Negative Affect, a paired samples *t*-test compared baseline and post-extinction CS+/CS- differentials.

The association between SCRs and US-expectancy ratings was assessed with multilevel models run in R (nlme package). First, models using the overall phase means were run separately for acquisition and extinction with differential US-expectancies as the dependent measure and differential SCRs as the fixed effect. To examine early/late differences, we ran two more models using the early/late means with differential SCRs, Time (early/late) and SCR × Time interaction as fixed effects. For all models, participants were entered as random effects.

For the association between SCR and affective ratings, a multiple regression was conducted with differential SCRs during overall acquisition and extinction as predictors of the differential in post-extinction Negative Affect. The differential in baseline Negative Affect was added as a covariate. A similar multiple regression tested the effects of differential US-expectancy ratings on differential post-extinction Negative Affect. To examine the effects at specific sections of the task, the models were also run with differential SCRs or US-expectancy ratings at early and late acquisition and extinction as predictors of the differential in post-extinction Negative Affect.

For all models the p-value threshold was adjusted with Bonferroni correction to control for eight independent tests (adj. p-value = .006). Analyses were conducted on the full sample, including both contingency aware and unaware participants. Running the analyses with contingency aware individuals only resulted in similar findings, thus we present data for the larger sample.

## 3 Results

### 3.1 Descriptive analyses

**Fig. 2:**
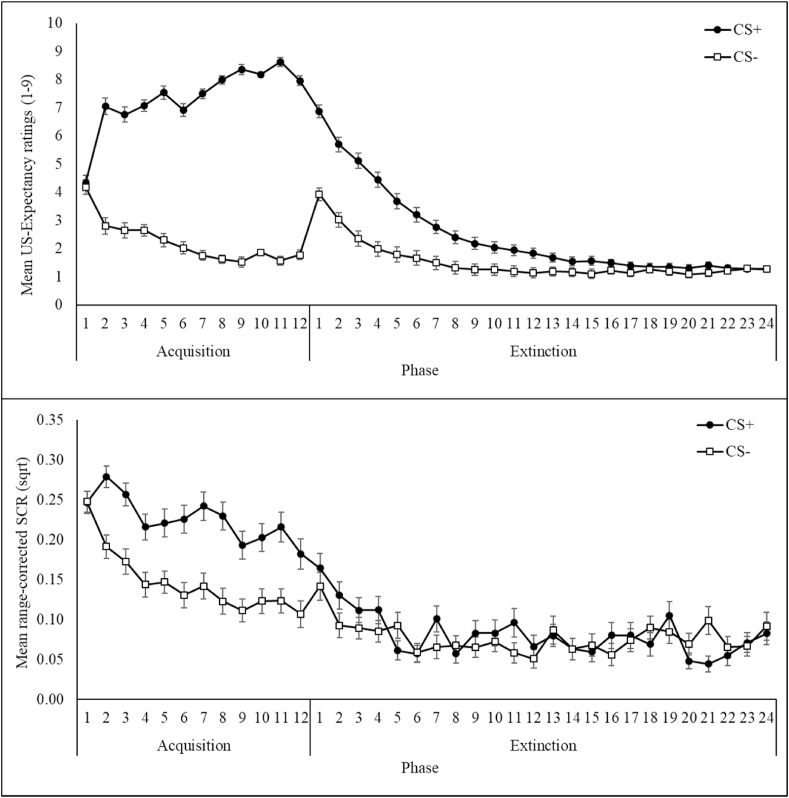
Mean US-expectancy ratings (top panel) and mean SCRs (bottom panel) for each CS separately at each trial of acquisition and extinction. Bars represent standard error of mean.

Fig. 2 illustrates the pattern of SCRs and US-expectancy ratings trial-by-trial throughout acquisition and extinction. Repeated measures ANOVAs indicated an expected Phase effect for both differential SCRs, F(1,72) = 55.79, p < .001, η_p_^2^ = 0.44, and differential US-expectancy ratings, F(1,72) = 487.38, p < .001, η_p_^2^ = 0.87. Specifically, CS discrimination in SCRs and US-expectancy ratings was significantly higher during acquisition than during extinction.

Furthermore, a significant Phase × Time interaction was also observed for both SCRs and US-expectancy ratings, F(1,72) = 6.49, p = .01, η_p_^2^ = 0.08, and F(1,72) = 241.39, p < .001, η_p_^2^ = 0.77, respectively. Follow-up analyses showed that for SCRs, there was a significant decrease in the CS+/CS- differential in late (M = −.01, SD = 0.07) compared to early extinction (M = 0.01, SD = 0.06), t(72) = 2.51, p = .01, but early (M = 0.06, SD = 0.08) and late (M = 0.08, SD = 0.10) acquisition did not differ significantly. For US-expectancy ratings, the CS+/CS- differential was significantly higher at late (M = 6.59, SD = 1.43) than early (M = 3.22, SD = 1.83) acquisition, t(72) = -13.07, p < .001, but significantly reduced at late (M = 0.14, SD = 0.57) compared to early extinction (M = 2.07, SD = 1.88), t(72) = 9.07, p < .001.

For post-extinction Negative Affect, a paired samples *t*-test showed significantly higher CS+/CS- discrimination after extinction (M = 2.99, SD = 2.17) in comparison to baseline (M = −0.02, SD = 1.38), t(71) = -9.67, p < .001. These analyses overall indicated that differential fear learning was successfully achieved and subsequently extinguished, although Negative Affect CS+/CS- discrimination was present at the end of extinction.

### 3.2 Association between SCR and US-expectancy ratings

Pearson's bivariate correlations using overall phase averages indicated small and non-significant associations between differential SCRs and differential US-expectancy ratings (acquisition: r(73) = 0.14, p = .24; extinction: r(73) = 0.21, p = .07).

**Fig. 3:**
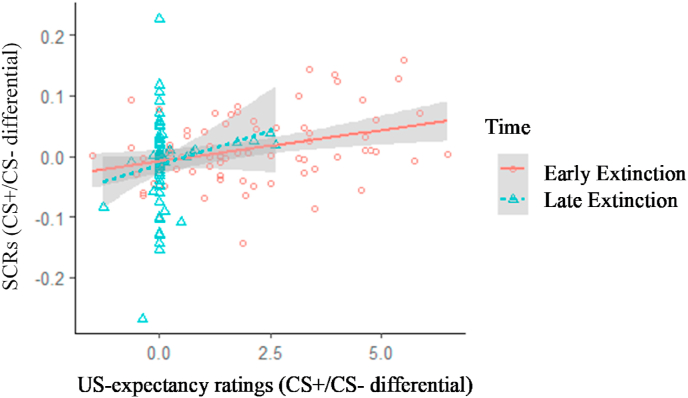
SCR x Time (Early vs. Late) interaction on US-expectancy ratings during fear extinction.

However, examining early and late acquisition and extinction showed moderate positive associations between differential SCRs and differential US-expectancy ratings for the early part of each phase, albeit not reaching Bonferroni-corrected significance. Table 1 illustrates correlations among all outcome measures using early/late averages (p-values were Bonferroni adjusted for 18 correlations). These associations were further explored with two multilevel models summarized in Table 2. Model 1 showed that the CS+/CS- discrimination in SCRs was positively associated with CS+/CS- discrimination in US-expectancy ratings throughout acquisition, as there was no significant SCR × Time interaction. Similarly, for extinction (Model 2), CS+/CS- discrimination in SCRs was significantly positively associated with discrimination in US-expectancy ratings. The SCR × Time interaction failed to reach Bonferroni-corrected significance (p = .014), but in an exploratory manner, we ran separate models for early and late extinction. These showed that the effect of SCRs on US-expectancy ratings was significant at early extinction (Beta = 10.68, S.E. = 3.61, t(71) = 2.96, p = .004) only. As Fig. 3 illustrates, differential US-expectancy ratings reach floor effects by late extinction, which may explain the lack of associations in this section.

**Table 1:** Bivariate correlations among differential SCRs and differential US-expectancy ratings during early and late fear acquisition and extinction, and correlations of both with differential post-extinction Negative Affect.

		US-expectancies (CS+/CS- differential)				
**SCRs (CS+/CS- differential)**		Early Acquisition	Late Acquisition	Early Extinction	Late Extinction	Post-extinction Negative Affect
	Early Acquisition	.30				.10
	Late Acquisition		.23			.18
	Early Extinction			.33^†^		.09
	Late Extinction				.18	-.12
**Post-extinction Negative Affect (CS+ /CS- differential)**		.01	.17	.27	.17	

Note: ^†^p < .005.

**Table 2:** Multi-level models examining the effect of SCRs (CS+/CS- differential), Time (early vs. late) and their interaction on US-expectancy ratings (CS+/CS- differential) during fear acquisition (Model 1) and extinction (Model 2).

	Model 1- Acquisition, DV: US-expectancies (CS+/CS- differential)			Model 2- Extinction, DV: US-Expectancies (CS+/CS- differential)		
Predictors	Beta	S.E.	95% CI	Beta	S.E.	95% CI
**Fixed effects**						
Intercept	2.81^c^	0.23	2.36/3.26	1.94^c^	0.16	1.63/2.25
SCR (CS+/CS- differential)	6.82^a^	2.20	2.49/11.15	10.46^b^	2.66	5.23/15.69
Time (Early vs. Late)	3.45^c^	0.31	2.83/4.06	−1.78^c^	0.21	−2.20/-1.93
SCR x Time	−2.77	2.86	−8.39/2.86	−8.71	3.44	−15.47/-1.93
**Random effects**						
Intercept	0.66			0.43		
Residual	1.42			1.23		
**Marginal R**^**2**^	0.56			0.40		

a p < .006.

b p < .00125.

c p < .000125.

### 3.3 Association between SCR and post-extinction Negative Affect

Pearson's bivariate correlations showed that differential SCRs during acquisition and extinction did not correlate significantly with differential post-extinction Negative Affect, r(73) = 0.19 and r(73) = -0.04 respectively. Similar small and non-significant associations (correlation coefficients ranging from 0.09 to 0.18) were observed when examining early/late parts of each phase (Table 1).

### 3.4 Association between US-expectancy ratings and post-extinction Negative Affect

Finally, differential US-expectancy ratings during overall acquisition were not significantly associated with post-extinction Negative Affect, r(73) = 0.16, p < .2, but differential US-expectancy ratings during overall extinction showed a moderate positive correlation, r(73) = 0.35, p = .003. These correlations were further explored with a multiple regression (Table 3), which predicted 11% of the variance, but did not reach Bonferroni-corrected significance, F(3,71) = 3.90, p = .012. Looking at individual predictors, higher CS discrimination in US-expectancies during extinction predicted higher CS discrimination in post-extinction Negative Affect (β = 0.34, p = .004).

**Table 3:** Multiple regression conducted on post-extinction Negative Affect (CS+/CS differential) with average US-expectancy ratings (CS+/CS differential) during overall acquisition and extinction as predictors. Baseline Negative Affect (CS+/CS differential) was included in the analysis as a covariate.

	Post-extinction Negative affect (CS+/CS differential)			
Predictors	B	S.E. B	β	95% CI
Constant	1.126	1.059		−0.99/3.24
Baseline Negative Affect	−.156	.178	-.099	−0.51/0.20
US-Expectancies diff. Acquisition	−.228	.200	.130	−0.17/0.63
US-Expectancies diff. Extinction	.749	.249	.339^b^	0.25/1.25
Model summary				
Adj. R^2^	.11			
F (df)	3.90 (3,71) a			

a p < .0125.

b p < .006.

Analyses with early/late averages for each phase indicated a similar pattern of modest associations between differential US-expectancy ratings at early extinction and differential post-extinction Negative Affect (Table 1), which did not reach Bonferroni-corrected significance.

## 4 Discussion

The present study examined associations among indices typically used in human fear conditioning research to assess learned fear. Although this is not a new question, recent theoretical debates indicate the need for further empirical studies of these associations (Fanselow & Pennington, 2018; LeDoux & Pine, 2016). This paper uses a correlational approach to explore the relationships among autonomic arousal and self-reports of associative and evaluative learning.

Findings indicate moderate positive associations among outcome measures of fear learning, suggesting that the various components of fear may be more integrated than sometimes thought (Lang, 1971; LeDoux & Pine, 2016). However, the strength of associations varied depending on the measures involved and the part of the fear learning process. Specifically, physiological (SCRs) and self-report (US-expectancy ratings) measures of associative learning were positively associated at early and late acquisition and early extinction. On the other hand, US-expectancy ratings during overall extinction were significantly associated with post-extinction negative affect, an evaluative learning measure. No associations between SCRs and post-extinction negative affect were observed.

The association among SCRs, the most frequently used physiological index of human fear conditioning, and US-expectancy ratings confirms prior literature suggesting that skin conductance reflects cognitive/associative aspects of fear conditioning (Dawson, Rissling, et al., 2007; Hamm & Vaitl, 1996; Lovibond, 1992; Weike et al., 2007). It is also consistent with the view of a shared learning process giving rise to independent conditional responses (single-process models; Lovibond & Shanks, 2002; Fanselow & Pennington, 2018). Correlations in our study were moderate, but substantial, given the moderate reliability of skin conductance in fear conditioning studies (ICC = 0.43 for test retest (12 weeks) reliability, Zeidan et al., 2012).

However, these associations were observed only when examining early/late sections of acquisition and extinction and specifically during acquisition and early extinction. Late extinction showed no associations, which can be attributed to the lack of response variance (the differential in US-expectancies reached zero) by the end of a long extinction procedure. Hence, we cannot make firm conclusions about associations among measures at this part of the extinction process. However, the fact that SCRs correlate with US-expectancies during acquisition and early extinction, fits with prior studies showing associations among SCRs and other measures like pupillary responses (Jentsch et al., 2020) during acquisition and amygdala activation during acquisition and early extinction (Phelps, Delgado, Nearing, & LeDoux, 2004). Our findings indicate that associations are not limited among physiological signals, but are also seen for US-expectancies and support the notion of coherence among responses during fear learning (acquisition) or relearning (early extinction).

As for the association between the two associative learning measures and post-extinction negative affect, this varied according to modality. SCRs were not correlated with negative affect, which may reflect differences between the two measures in timing (during acquisition/extinction vs. after extinction) and in modality. This lack of association, though, is not surprising since skin conductance, as an index of autonomic arousal, reflects the significance or relevance of a stimulus (here whether it will be followed by something “bad”), but not necessarily its unpleasantness (Lang, Greenwald, Bradley, & Hamm, 1993). As for US-expectancy ratings, these correlated moderately with post-extinction negative affect. As post-extinction negative affect has shown moderate test-retest (1 week) reliability (ICC = 0.37; Purves et al., 2019), current associations reflect a relatively substantial correspondence between associative and evaluative learning self-reports, supporting previous findings (Blechert et al., 2008). However, associations were significant only for US-expectancy ratings averaged across overall extinction. While associations of US-expectancies with SCRs were stronger when looking specifically at early extinction, associations with post-extinction negative affect were weaker. This difference may be because the affective ratings were collected after extinction. Thus, there is a temporal difference between the early learning stage of extinction and the negative affect assessment, which may have dampened the associations. Previous studies indicate that online affective ratings differ from post-extinction ones (Lipp et al., 2003) and when measured concurrently, affective ratings correlate strongly with US-expectancies (r's between 0.60 and 0.75; Mertens et al., 2018).

Although not as strong, our reported associations provide a noteworthy finding, i.e. that persistent US-expectancy to the CS+ throughout extinction is predictive of persistent negative affect to the CS+. Persistent post-extinction negative affect is an index with clinical relevance, as it has been associated with return of fear after extinction (Dirikx, Hermans, Vansteenwegen, Baeyens, & Eelen, 2004, 2007; Hermans et al., 2005; Zbozinek, Hermans, Prenoveau, Liao, & Craske, 2015). As a significant proportion of patients relapse after successful exposure therapy (Craske & Mystkowski, 2006), understanding the factors underlying the return of fear can assist in reducing relapse and increasing exposure treatment efficacy. Current findings indicate that one such factor may be the persistence of learned associations despite the experience of no adverse outcomes during extinction. Further research needs to explore this and other possible predictors of unextinguished negative affect.

Besides clinical implications, findings from this study have methodological implications for the assessment of learned fear. The correspondence among US-expectancy ratings and SCRs during fear acquisition and early extinction provides further evidence for the usefulness of US-expectancy ratings as an index of both fear and extinction learning (see Boddez et al., 2013 for the validity of US-expectancies). As fear conditioning paradigms are being adapted for use outside of the laboratory (Purves et al., 2019), and without the inclusion of standard physiological assessments, valid self-report measurements become even more crucial as measures of fear learning. Current findings also show a correspondence between US-expectancies and affective ratings. However, the moderate associations, along with recent findings stressing the importance of affective ratings (Mertens et al., 2018), suggest that a thorough assessment of fear learning requires both associative and evaluative learning indices.

Finally, some limitations of the study should be noted. Firstly, as mentioned above, affective ratings were collected only after extinction, to avoid additional exposure to the CSs before fear extinction (Lonsdorf et al., 2017). This may have influenced the associations under study. Additional affective ratings, e.g. post-acquisition or throughout each phase, would allow us to assess the pattern of evaluative learning self-reports and their association with other measures more accurately. Furthermore, the context change between fear acquisition and extinction may have influenced self-reports and physiological measures in different ways (Sjouwerman et al., 2015). Additionally, fear acquisition was partially instructed and instructions may alter the pattern of responding differentially for associative and evaluative measures (Mertens et al., 2016; Raes, De Houwer, De Schryver, Brass, & Kalisch, 2014). Another limitation is the lack of fear-potentiated startle, a physiological index considered to indicate fear specifically rather than general arousal (Grillon & Baas, 2003). The fear-potentiated startle correlates poorly with SCRs (Weike et al., 2007) and shows a different pattern than US-expectancy ratings (Hamm & Vaitl, 1996; Sevenster et al., 2012; 2014). In addition, we did not assess behavioural avoidance, a clinically relevant measure. The limited studies assessing behavioural avoidance during fear conditioning suggest that it correlates with indices of learned fear, like fear-potentiated startle (Cornwell, Overstreet, Krimsky, & Grillon, 2013), as well as persistent fear after extinction in clinical groups (Blechert et al., 2007). Future research could examine associations including these measures too.

## 5 Conclusions

This study illustrates that physiological and self-report measures of associative learning correlate moderately with each other during fear acquisition and early extinction. This further supports the use of US-expectancy ratings as a reliable index of the associative aspects of learned fear. US-expectancy ratings during extinction were also moderately related to self-reports of evaluative learning, but capture only a small portion of the evaluative aspects of fear. Thus, current findings support the use of multiple outcome measures in human fear conditioning research to assess fully all components of learned fear. Furthermore, examining these measures in conjunction using correlational approaches allows novel investigations of the fear learning process and can provide valuable information about the development and treatment of fear and anxiety.

## Appendix A Supplementary data

Application Application

Application Application

## Footnotes

1 Missing expectancy ratings were imputed using the average of the participant's previous and next rating for the same stimulus or the sample mean if the missing value occurred at the first trial. For missing values at the last trial, the participant's previous rating for the same stimulus was carried forward. In acquisition, 21 participants had at least one missing value (maximum per person: 3 out of 24), with 27 values imputed in total. In extinction, 46 participants had at least one missing value (maximum per person: 14 out of 48), with a total of 90 values imputed.

2 Exploratory factor analyses (EFA) were computed for CS+ and CS- ratings at each time point and indicated only one factor with eigenvalue > 1. For baseline CS+, the factor explained 64.5% of the variance (factor loadings 0.68, 0.80, 0.91), while at post-extinction the factor explained 79.4% of the variance (factor loadings 0.86, 0.88 and 0.93). For CS-, the factor explained 56.7% of the variance at baseline (factor loadings 0.49, 0.91, 0.80) and 72.3% of the variance at post-extinction (factor loadings 0.77, 0.86, 0.91).
