# Supplementary material for: Measuring fear: Association among different measures of fear learning
Source: J Behav Ther Exp Psychiatry. 2021 Mar;70:101618. doi: 10.1016/j.jbtep.2020.101618 (PMC7689577; doi:10.1016/j.jbtep.2020.101618)
Supplement: Application [file mmc1.docx]

**Supplemental Material**

**Methods**

**Participant exclusions:**

The data from three participants were unusable (two due to technical issues and one due to problematic electrode placement and the participant falling asleep). Furthermore, four participants were excluded as extreme outliers: three participants provided the exact same US-expectancy rating across all trials at either acquisition or extinction (5 or 1) and one participant provided the exact same value at all affective ratings (9), as well as US-expectancy ratings between 5-7 at all extinction trials. Including these four participants in the analyses does not alter the results presented in the paper.

**Shock Calibration procedure:**

During the calibration procedure, electric shocks of incrementing intensity were delivered and participants rated each shock on a 10-point Likert scale (1= “completely not painful, didn’t even feel it”, 10= “the most painful it could be”). Calibration started at 10V with a 10V increment per step, which reduced to a 5V increase per step when participants mentioned feeling the shock, and then further reduced to 2.5V per step when approaching the desired level. Intensities were increased up to the level that participants perceived as *“unpleasant, but not yet painful”* (estimated at a rating of 7 out of 10). Average intensity selected by participants was 53.20V (SD=12.18, range=25-75). Post-acquisition unpleasantness ratings for the shocks confirmed the intended unpleasantness level, with average unpleasantness of 7.36 (SD=0.98, range=4-10) on a 10-point Likert scale (1= ‘Not unpleasant at all’, 10= ‘Very unpleasant’).

**Fear acquisition - Trial randomization:**

Participants were randomly allocated to four different trial orders created with the following parameters:

(a) the first two trials were a CS+ and a CS-

(b) the first CS+ was always paired with the shock and

(c) there were no more than two consecutive trials with the same CS.
